# Encouraging Behavior Changes and Preventing Cardiovascular Diseases Using the Prevent Connect Mobile Health App: Conception and Evaluation of App Quality

**DOI:** 10.2196/25384

**Published:** 2022-01-20

**Authors:** Dahbia Agher, Karima Sedki, Sylvie Despres, Jean-Pierre Albinet, Marie-Christine Jaulent, Rosy Tsopra

**Affiliations:** 1 Inserm, University Sorbonne Paris Nord, Sorbonne University Laboratory of Medical Informatics and Knowledge Engineering in e-Health, LIMICS Paris France; 2 BeWellConnect Research and Development, Visiomed Group Puteaux France; 3 Inserm, Université de Paris, Sorbonne Université, Centre de Recherche des Cordeliers, F-75006 Paris France; 4 HEKA, Inria Paris France; 5 Department of Medical Informatics, Hôpital Européen Georges-Pompidou, Assistance Publique - Hôpitaux de Paris Paris France

**Keywords:** digital health, mHealth, mobile application, IT, technology, prevention, cardiovascular risk factor, behavior change, primary care

## Abstract

**Background:**

Cardiovascular diseases are a major cause of death worldwide. Mobile health apps could help in preventing cardiovascular diseases by improving modifiable risk factors such as eating habits, physical activity levels, and alcohol or tobacco consumption.

**Objective:**

The aim of this study was to design a mobile health app, Prevent Connect, and to assess its quality for (1) assessing patient behavior for 4 cardiovascular risk factors (unhealthy eating, sedentary lifestyle, alcohol, and tobacco consumption) and (2) suggesting personalized recommendations and mobile health interventions for risky behaviors.

**Methods:**

The knowledge base of the app is based on French national recommendations for healthy eating, physical activity, and limiting alcohol and tobacco consumption. It contains a list of patient behaviors and related personalized recommendations and digital health interventions. The interface was designed according to usability principles. Its quality was assessed by a panel of 52 users in a 5-step process: completion of the demographic form, visualization of a short presentation of the app, testing of the app, completion of the user version of the Mobile App Rating Scale (uMARS), and an open group discussion.

**Results:**

This app assesses patient behaviors through specific questionnaires about 4 risk factors (unhealthy eating, sedentary lifestyle, alcohol, and tobacco consumption) and suggests personalized recommendations and digital health interventions for improving behavior. The app was deemed to be of good quality, with a mean uMARS quality score of 4 on a 5-point Likert scale. The functionality and information content of the app were particularly appreciated, with a mean uMARS score above 4. Almost all the study participants appreciated the navigation system and found the app easy to use. More than three-quarters of the study participants found the app content relevant, concise, and comprehensive. The aesthetics and the engagement of the app were also appreciated (uMARS score, 3.7). Overall, 80% (42/52) of the study participants declared that the app helped them to become aware of the importance of addressing health behavior, and 65% (34/52) said that the app helped motivate them to change lifestyle habits.

**Conclusions:**

The app assessed the risky behaviors of the patients and delivered personalized recommendations and digital health interventions for multiple risk factors. The quality of the app was considered to be good, but the impact of the app on behavior changes is yet to be demonstrated and will be assessed in further studies.

## Introduction

Cardiovascular diseases (CVDs) are a major cause of death worldwide [1] for which several causal risk factors have been identified [2]. One of the keys to prevent CVD is reducing the impact of modifiable risk factors such as unhealthy eating, physical inactivity, alcohol consumption, and smoking [3-7]. This can be achieved by providing advice and encouraging changes in patient behavior, particularly as concerns these risk factors. Mobile health (mHealth) interventions [8-10] can be useful for improving lifestyle behaviors relating to CVDs. mHealth technology is defined as mobile devices such as mobile phones, patient monitoring devices, personal digital assistants, or other wireless devices intended to be worn, carried, or accessed by patients or health care providers to monitor health status or improve health outcomes [3,4].

mHealth interventions may take various forms, including SMS text messages, interactive voice responses, surveys, focus groups, smartphone health apps, and solutions combining several aspects of connected health. Many of these interventions have been shown to have a significant impact on the management and improvement of modifiable risk factors in CVD prevention [5-7,11-14]. For example, many studies [14] have reported promising results for mHealth interventions in the improvement of patient outcomes such as body measurements (eg, weight, waist circumference), metabolic and physiological measurements (eg, blood pressure, glucose levels), adherence to and safe use of medication, physical activity performance, meal management, and awareness of health conditions and treatment options.

Despite the emergence of mHealth and research into lifestyle behavior changes, most of the interventions developed to date target one single risk factor for CVD prevention. Positive effects of mHealth interventions have been demonstrated for single risk factors for CVD, for example, Text2Quit [15] for smoking cessation, ASA24 [16] for recording food intake, and mDiet [17] for weight loss and for alcohol intake [18]. Some studies [14] have also examined the impact of mHealth interventions on multiple risk factors, but these studies have reported only limited success. These studies were limited by a lack of personalization for either delivering recommendations or for selecting the most appropriate form of intervention (SMS, email, etc) as a function of patient profile. Furthermore, many of the apps are at risk of becoming rapidly obsolete owing to the fast pace at which technologies are progressing, and new technological innovations must therefore be considered. For example, the latest mobile technologies can connect and interact with each other, update, and track personal health data in real time, and send alerts to users. In addition, the number of users with a modern smartphone has considerably increased in recent years (from 35% in 2011 to 81% in 2019 [9]). Likewise, most health apps have encountered serious usability problems [19] or have not undergone usability assessment [20]. Usability affects the efficiency and efficacy of the app (eg, time to complete tasks, errors) [21] and must be considered to increase the chance of the app being successfully adopted by patients.

Here, we designed an mHealth app, Prevent Connect, for (1) assessing patient behaviors for 4 important risk factors (unhealthy eating, sedentary lifestyle, alcohol, and tobacco consumption) and (2) suggesting personalized recommendations and mHealth interventions for risky behaviors. We aimed to design an app with sufficiently high usability to favor its adoption by patients. We describe the Prevent Connect app and the evaluation of its quality and usability.

## Methods

### Design of Prevent Connect

#### Knowledge Base

The knowledge base of the mobile app was based on French national recommendations for healthy eating, physical activity levels, and limiting alcohol and tobacco consumption [22-25]. We analyzed these recommendations and extracted the necessary variables for the assessments of each behavior (eg, for healthy eating, we extracted 23 variables, each with 7 possible answers). We then combined the variables and their responses to establish a list of patient behaviors. For each patient behavior, we calculated a qualitative score extending from “very good behavior” to “very bad behavior” (based on guidelines). We then associated personalized recommended actions with these behavior scores (eg, “do the equivalent of 30 minutes of physical activity per day”) and digital health interventions (eg, “activity tracker for self-monitoring of daily activity”). The identified patient behaviors and their associated qualitative scores for risky behavior, personalized recommendations, and digital health interventions were then implemented within the app.

#### Interface

The mobile app was ergonomically designed according to usability principles [26,27], and the graphical interface was implemented with Gluon mobile technology [28]. The navigation system was based on a 3-step process: (1) assessment of each risk factor with specific questionnaires, (2) automatic assessment, by the app, of risky behavior, and visualization of recommended and personalized actions, and (3) suggestions for digital health interventions. The interface was designed according to the following usability principles [26,27]: simplicity, naturalness, and effective use of language by using concise, appropriate, and understandable language; consistency, minimizing cognitive load, and efficient interactions by dividing the navigation into a 3-step process and adding a clear taskbar and meaningful colors; and effective information presentation, by limiting the amount of text and using comprehensible icons (eg, a wineglass icon was used to represent alcohol consumption). The consideration of usability principles in app design can help reduce the user’s cognitive workload and increase confidence in the app [29].

### Evaluation of Prevent Connect

#### General Study Design and Ethics Approval

We carried out an evaluation of the quality of the app by asking patients identified as potential future users to test the app. We organized 20 online sessions, each with 1-5 users. Each session began with a demonstration of the app and then testing of the app by the study participants. The opinions of the study participants were then collected in an electronic form and through an open discussion. The study protocol was validated by the appropriate Inserm ethics committee (CD/EB 20-023, 20-660, IRB000388, IORG0003254, FWA0005831).

#### Recruitment 

Study participants were recruited online via a dedicated website and by word-of-mouth from April 15 to June 15, 2020. For participation, subjects had to be older than 18 years and not treated for CVD (eg, stroke, coronary artery disease). They also had to sign an online consent form and agree to participate without compensation.

#### Study Design and Statistical Analysis

The evaluation was performed online and lasted from 30 minutes to 1 hour, depending on the session. It proceeded via 5 successive steps:

1. In step 1, study participants completed an online form to provide sociodemographic information. Questions were adapted so as to guarantee anonymity, and no personal information was recorded (eg, participants were asked to indicate their age group rather than an exact age to limit the risks of reidentification).
2. In step 2, study participants watched a short demonstration of the app.
3. In step 3, study participants used and tested the app on their own. They first completed the questionnaires for behavior assessment and visualized the recommendations delivered by the app.
4. In step 4, study participants completed the user version of the Mobile App Rating Scale (uMARS) evaluation form online, together with 2 additional questions about satisfaction and possible areas of improvement. The uMARS form contains 26 items for assessing app quality [30]: 5 items for app engagement, 4 items for app functionality, 3 items for aesthetics, 4 items for app information, 4 items for the subjective quality of the app, and 6 items for the perceived impact of the app. The global quality of the app was then assessed by calculating the mean score for the uMARS items corresponding to the categories engagement, functionality, aesthetics, and information, according to the following formula [31]: mean score = (engagement score + functionality score + aesthetics score + information score)/4, with a maximum mean score of 5.
5. In step 5, study participants were invited to discuss the app freely in groups. They were allowed to give their opinions freely and to discuss the app together.

For sociodemographic data and for each item of the uMARS scale, we calculated the percentage of each response. For the free discussion, data were analyzed, broken up, and combined into similar themes. Each theme was mapped to one of the categories of the uMARS scale (eg, engagement, functionality).

## Results

### The Prevent Connect App

**Figure 1:**
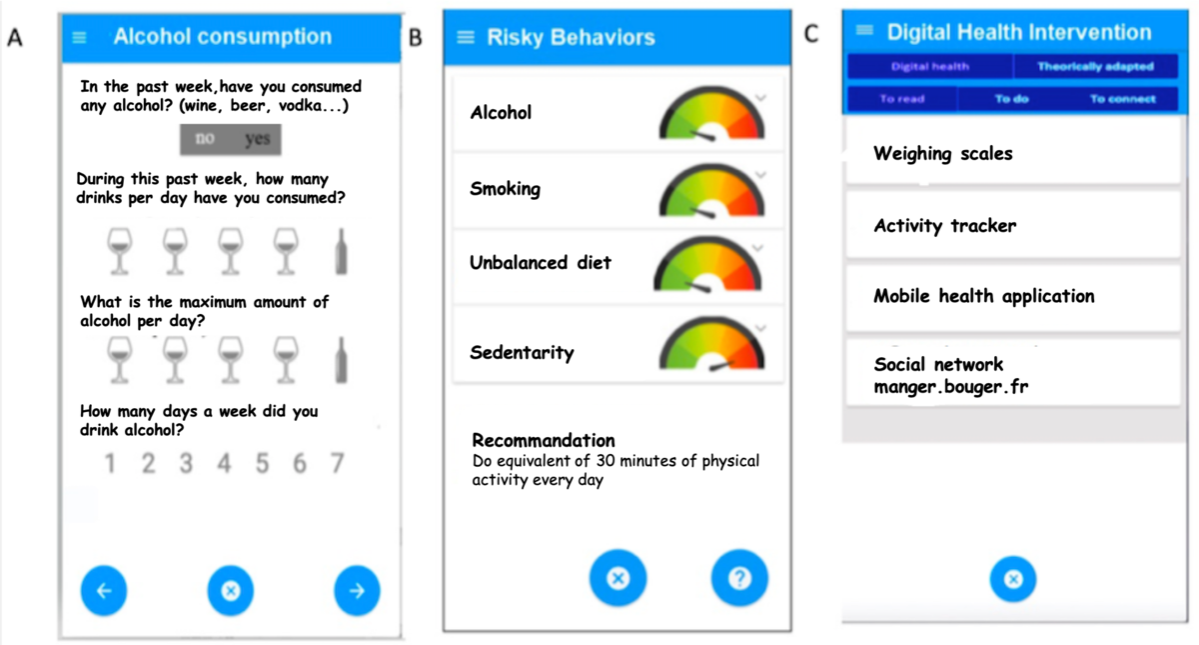
Illustration of the “Prevent Connect” app. On the first interface, users complete questionnaires to assess their behavior for each risk factor (eg, in area A, the alcohol consumption questionnaire is shown). The app automatically identifies risky behaviors and the users can consult this result and the recommended actions on the second interface (eg, in area B, the user is sedentary, and a personalized recommendation is displayed). On the last interface, users can consult a list of targeted digital interventions that are personalized according to their risky behaviors (eg, in area C, an activity tracker or a weighing scale is suggested because the patient is sedentary). Note: manger.bouger.fr is a well-known social network in France that provides additional tips and advice regarding physical activities and healthy eating.

Use of the app begins with the login interface (Figure 1). Once the users have logged in, they visualize the home interface, which includes several tabs (eg, welcome first time, behavior questionnaire). During the first connection, patients are asked a number of questions about themselves (eg, gender, age, family history, social context) and behaviors concerning eating habits, physical activity, and tobacco and alcohol consumption. Once these forms have been completed, the app provides a recap of the behavior assessment for each risk factor in the form of a gauge with an arrow indicating the position of the user on a color scale extending from green for “very good behavior” to red for “very bad behavior” (graduated scale depending on the qualitative score calculated for risky behavior). Personalized and recommended actions regarding behavior changes are also displayed at the bottom of the interface. Users then go on to the next screen, which displays the personalized digital health interventions that would be helpful in encouraging behavior changes. A help icon is also provided to allow users to obtain additional explanations or information on request.

### Evaluation of the Quality of the Prevent Connect App

#### Description of the Panel of Study Participants

In total, 52 individuals tested and evaluated the app: 28 women and 24 men. The study participants were aged between 18 and 69 years and had diverse professional profiles. Most participants were familiar with digital devices and used a smartphone, computer tablet, or laptop computer at least once per week. Half of the participants declared that they had already used a connected object, mostly with an app, but only 13% (7/52) declared that they had already assessed their cardiovascular risk in this way (Table 1).

**Table 1:** Sociodemographic characteristics of the study participants (N=52).

Characteristics		Values, n (%)
**Sex**		
	Female	28 (54)
	Male	24 (46)
**Age (years)**		
	18-24	8 (15)
	25-34	23 (44)
	35-49	16 (31)
	50-69	5 (10)
**Socioprofessional category**		
	Student	11 (21)
	Executive	24 (46)
	Employee	2 (4)
	Liberal profession	9 (17)
	Unemployed	4 (8)
	Retired	2 (4)
**Use of smartphone, laptop computer, or computer tablet at least once per week**		
	Smartphone only	4 (8)
	Laptop computer only	2 (4)
	Computer tablet only	0 (0)
	Smartphone and laptop computer	29 (56)
	Smartphone and computer tablet	2 (4)
	Smartphone, computer tablet, and laptop computer	15 (29)
**Use of connected objects (eg, activity tracker)**		
	Yes, at least once per day	10 (19)
	Yes, at least once per week	7 (13)
	Yes, at least once per month	4 (8)
	Yes, less than once per month	6 (12)
	No use	25 (48)
**Mode of connected object use**		
	Exclusively with the help of a health professional	1 (2)
	Exclusively with an app	23 (44)
	By turning the Wi-Fi off	1 (2)
	With a health professional and app	2 (4)
	No use	25 (48)
**Evaluation of cardiovascular risk (present or past)**		
	Yes, less than once per month	7 (13)
	No	45 (87)
**Mode of cardiovascular risk evaluation**		
	Health professional	3 (6)
	Apps	2 (4)
	Website	1 (2)
	Score	1 (2)
	No item	45 (86)

#### App Quality

**Figure 2:**
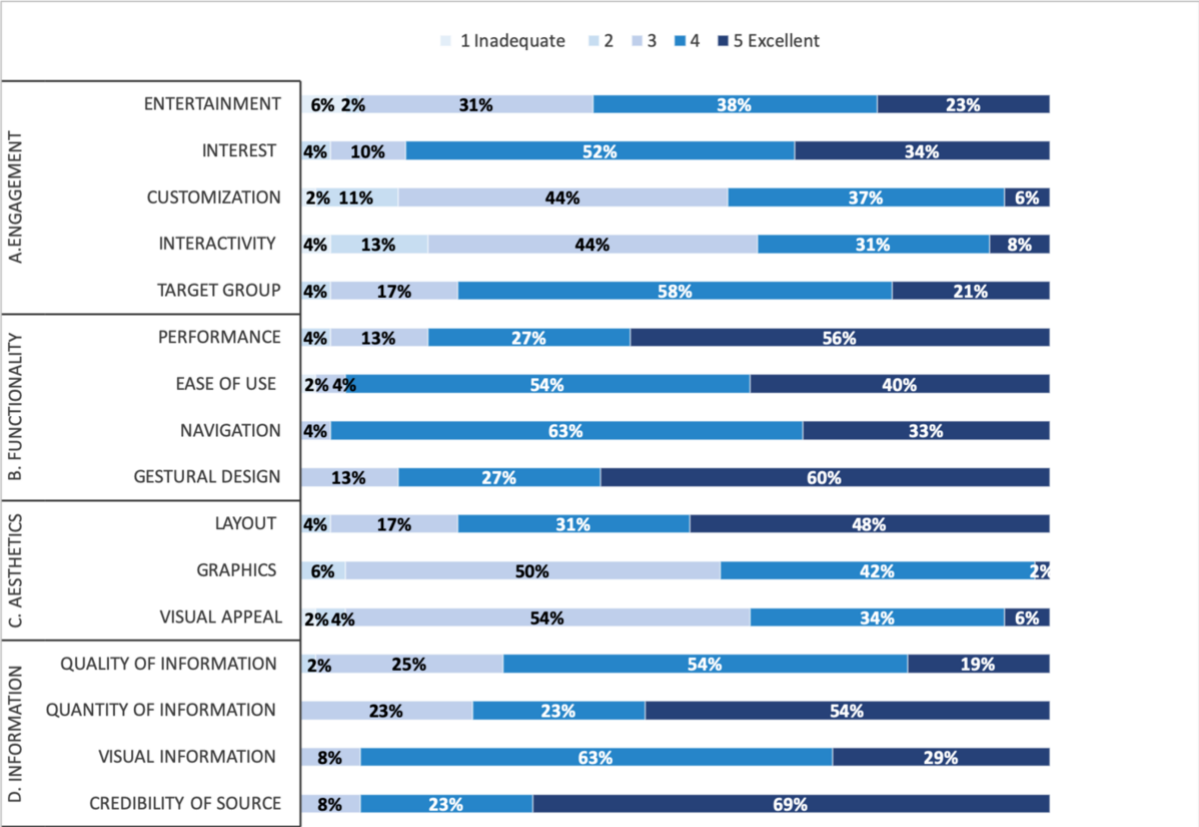
Distribution of users’ responses to the user version of the Mobile App Rating Scale (N=52, except for the item “credibility of source,” for which only 51 answers were available, and for the item “visual information,” for which only 48 answers were available).

The mean total score for app quality was 4 on the uMARS scale (maximum mean score of 5, Figure 2).

For *engagement*, the mean uMARS score was 3.7 (SD 0.7). More than three-quarters of the study participants found the app interesting and appropriate for the target audience, and more than half found it fun and entertaining (Table 2, comment 1). One study participant said that the app was appropriate and accessible to everyone, including older adults who were less used to apps (Table 2, comment 2). However, fewer than half of the study participants found the app sufficiently interactive and customizable. Some study participants suggested making the app more interactive by adding a chatbot and the possibility of speaking to an expert, together with additional reminders and alerts concerning the recommended behavior changes. A few study participants also suggested delivering a daily preventive message to help educate users (Table 2, comment 3).

For *functionality*, the mean uMARS score was 4.4 (SD 0.6). Almost all the study participants appreciated the navigation system and found the app easy to use. More than 80% of the participants also appreciated the performance and design of the app (Table 2, comment 4). Study participants said that they found the app intuitive and very easy to use, although 1 person suggested adding a tutorial (Table 2, comment 5). None of the study participants declared having experienced technical problems during the evaluation.

**Table 2:** Examples of feedback from study participants.

Section, subsection			Extract of comments	
**Engagement**				
	Entertainment and interest	…*The mobile app is fun and enables us to assess our behaviors.* [Comment 1]		
	Customization and interactivity	…*The app could be more interactive and customizable with a chatbot, the possibility to talk with an expert, but also with reminders and alerts about the recommended behavior changes.* [Comment 2]		
	Target group	…*The app is appropriate and accessible to everybody, even for older adults less accustomed to apps.* [Comment 3]		
**Functionality**				
	Performance	…*I didn't have any technical problems when I used the app.* [Comment 4]		
	Ease of use and navigation	…*Very practical and easy to use. The app can be used to make a quick assessment, but also encourages behavior changes, such as doing more sport or adapting food quantity.* [Comment 5]		
**Aesthetics**				
	Layout	…*The aesthetics could be improved by adding 3D pictures, video animations, summaries, pictograms (eg, arrows), and emojis.* [Comment 6]		
	Graphics, visual	…*Colors and graphics are consistent with a health app. Even if the blue color is currently used for health apps, I would prefer green*. [Comment 7]		
**Information**				
	Quality and quantity	…*The app content is of good quality (…). The content is right relevant, concise, interesting, clear and detailed.* [Comment 8]		
	Visual	…*Regarding app content, you could add shocking images and information like those displayed on cigarette packets or during the campaign for preventing traffic accidents.* [Comment 9]		
	Credibility of source	…*This app inspires more confidence than apps available on app stores.* [Comment 10]		
**Subjective**				
	Quality	…*Yes, this is a good app. I would give it a score of 4 out of 5, and I will use it to check my results and to see if I have achieved the target behaviors.* [Comment 11]		
**Perceived impact**				
	Awareness and knowledge	…*This is a very good app for becoming aware of your own behavior and changing your lifestyle, and thus for preventing health problems.* [Comment 12]		
	Seeking help and behavioral changes	…*Used alone no, but associated with follow-up by a health professional, why not?* [Comment 13]		

For *aesthetics*, the mean uMARS score was 3.7 (SD 0.6). More than three-quarters of the study participants found the layout appropriate. However, less than half felt that the graphics were of sufficiently high quality and visually appealing. Some study participants found the design and colors appropriate for a health app, whereas others felt that the colors were insufficiently attractive (Table 2, comment 7). Some study participants suggested improving the aesthetics of the app by adding more 3D pictures, video animations, summaries, pictograms, (eg, arrows), and emojis (Table 2, comment 6).

For *information*, the mean uMARS score was 4.2 (SD 0.5). More than three-quarters of the study participants found the app content appropriate, relevant, concise, and comprehensive (Table 2, comment 8). Study participants said they found the content interesting, clear, and detailed. Almost all the study participants also appreciated the visual elements used to display information and had confidence in the credibility of the source used to build the app content (Table 2, comment 9). One study participant said that this app inspired more confidence than those available on app stores (Table 2, comment 10). Some study participants suggested improving the questionnaires used to assess cardiovascular risk by adding more accurate questions about family history, tobacco and alcohol use, and physical activities, for example, or by adding questions about drugs and making the eating habit forms easier to complete. Other study participants suggested adding more information about dairy products, sports activities, and recipes. One study participant suggested adding explanations about the workings of the human body and physiology (eg, heart physiology).

**Figure 3:**
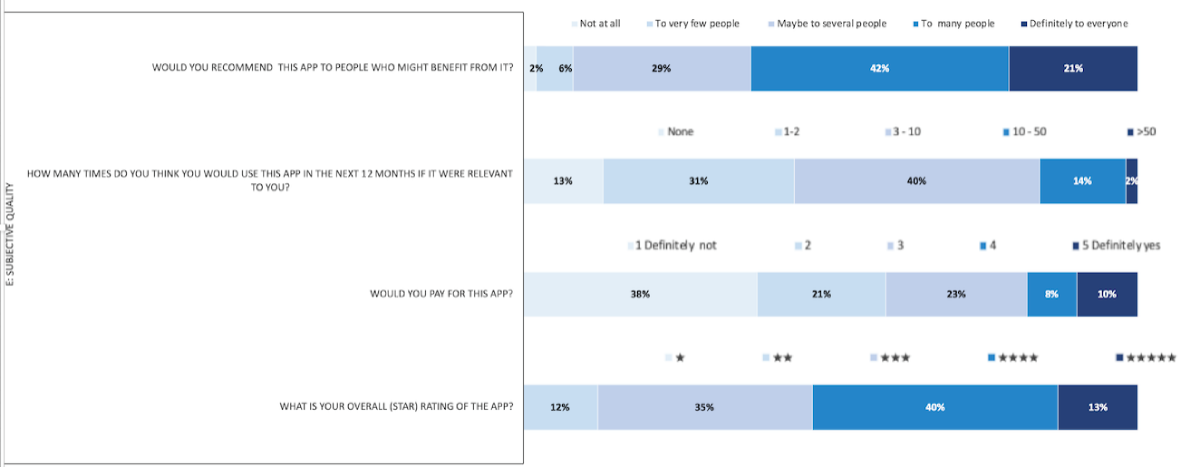
Distribution of users’ responses to the user version of the Mobile App Rating Scale for subjective quality (N=52).

For *subjective opinion* about app quality, 92% (48/52) of the study participants said they would recommend the app to several people, 56% (29/52) of the study participants also declared that they would use the app more than 3-10 times in the next 12 months, and 59% (31/52) said that they would not be willing to pay for it. Finally, 88% (46/52) of the participants gave more than 3 stars for the app when asked to rate it (Table 2, comment 11) (Figure 3).

#### Perceived Impact, Global Satisfaction, and Areas of Improvement

**Figure 4:**
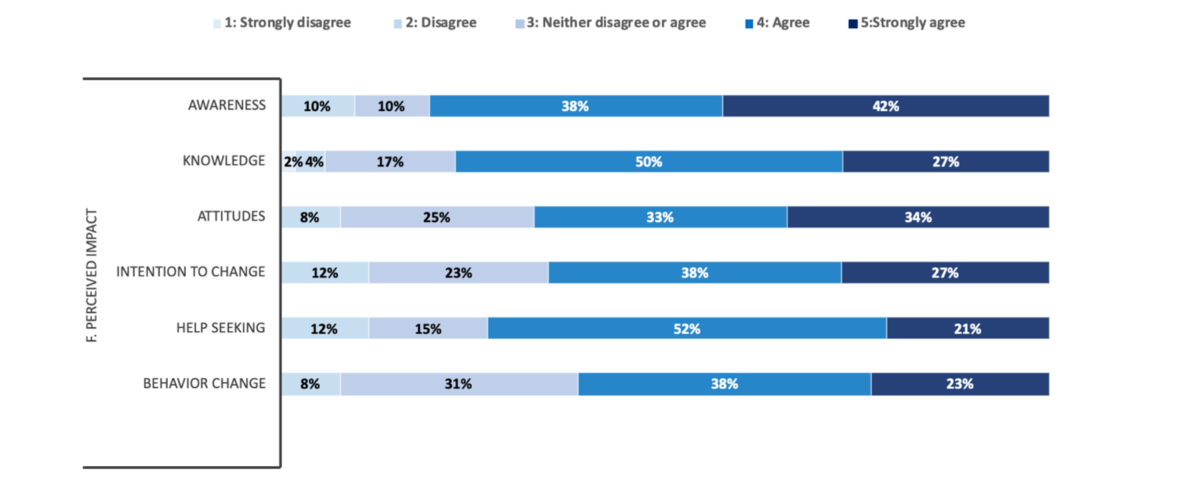
Distribution of users’ responses to the user version of the Mobile App Rating Scale for perceived impact (N=52).

For *perceived impact* (Figure 4), 80% (42/52) of the study participants declared that the app had made them more aware of the importance of addressing health behavior, and 77% (40/52) said that it had helped to improve their awareness of their behavior. Some study participants explained that the automatic assessment of behaviors had improved their understanding of their lifestyles and attitudes. One study participant also said that this awareness could encourage changes in behavior to prevent health disorders (Table 2, comment 12). Overall, 67% (35/52) of the participants said that the app had changed their attitudes toward improving health behavior, and 65% (34/52) said that they were more motivated to change their habits by playing more sports or eating more healthily, for example. One study participant said that the app could help him to achieve his personal goals in terms of behavior changes. Furthermore, 61% (32/52) of the participants thought that the app would lead to improvements in health behavior, and 73% (38/52) thought that the app would encourage people to seek further help if required (Table 2, comment 13).

For *global satisfaction*, 55% (29/52) of the study participants were fully satisfied with the app, and only 12% (6/52) were not. The others were neutral. Almost all the study participants found that the recommendations delivered were clear.

For *areas of improvement*, some study participants suggested extending the app to cardiovascular follow-up. They suggested adding new functionalities for tracing behavioral changes such as functions for recording meals or an activity tracker for recording physical activity. They also suggested displaying the data collected through their other connected objects (eg, sleep data collected with connected watch) within the app. One participant also suggested adding a virtual avatar to assess progress in behavioral changes over time.

## Discussion

### Principal Results

We designed Prevent Connect, an app based on national recommendations, to encourage behavioral change for CVD prevention. This app assesses 4 risk factors (unhealthy eating, sedentary lifestyle, alcohol, and tobacco consumption) and delivers personalized recommendations and digital health interventions to encourage changes in behavior. The quality and usability of the app were assessed by 52 potential future users. They considered the app to be of good quality, with a mean uMARS quality score of 4 on a 5-point Likert scale. The functionality and information content of the app were particularly well appreciated, with uMARS scores above 4. The aesthetics and the engagement of the app were also appreciated (uMARS score 3.7), but further improvements are required.

### Strengths and Limitations

This study has several strengths. First, the app is not limited to a single risk factor, but instead covers multiple risk factors. It also delivers recommendations and digital health interventions personalized according to the profile of the patient. Furthermore, the app is available for use on mobile phones, which should facilitate its adoption by patients [32,33]. Second, we considered usability principles [26,27] such as simplicity, naturalness, effective use of language, consistency, minimizing cognitive load, efficient interactions, and effective information presentation to improve the design of the app. It has been shown that the consideration of usability principles can considerably reduce user cognitive workload and increase confidence in technology [29], thereby increasing the likelihood of successful adoption of the app [34,35]. Third, we assessed the quality and usability of the app with a diverse panel of 52 potential users. We used the uMARS scale, which has been validated [31] for the assessment of various aspects of quality (engagement, functionality, aesthetics, and information), and we organized a free discussion with participants. The assessment of both quality and usability is a crucial step in the software lifecycle. Most health apps encounter serious usability problems [19], which may have a negative impact on app efficiency and efficacy (eg, time to complete the tasks, content errors) [21], thereby decreasing the likelihood of app adoption. Usability testing can identify any serious issues early in app development, making it possible to improve the app.

Our study also has several weaknesses. It focuses on primary rather than secondary prevention as it is vital to trigger behavioral changes early before the disease appears to prevent CVD [36]. However, we plan, in the future, to extend the scope of the app to secondary prevention. Another limitation is that we assessed only the quality of the app—not its impact on changes in behavior. Changing behavior is a complex process [37], and even if this app makes patients more aware of their risky behaviors and improves their understanding of healthy behavior, we cannot guarantee that this will be sufficient to have a positive impact on behavior. We plan to set up a randomized controlled trial to assess the impact of this app on changes in behavior and CVD prevention.

### Comparison With Other Studies

We propose here interventions for changing behavior at the individual level. Other interventions such as mass media campaigns have been proposed to induce changes in behavior at the population level [38]. They have the advantage of reaching a larger population through the dissemination of prevention messages via the mass media such as television, radio, or newspapers [38]. They also have the potential to produce positive behavior changes and to prevent negative changes [38]. However, they are not personalized to individuals and usually expose the population to the negative consequences of risky behavior, such as the arterial damage caused by smoking (Australian campaign “Every Cigarette is Doing You Damage” [39]). Traditional interventions are designed to evoke fear [32], but it is also important to deliver more positive messages [40]. By doing this, our app may help individuals to change their behavior with greater motivation and enthusiasm, favoring the persistence of behavioral changes over time [41]. Various apps have been developed for cardiovascular prevention at the individual level. However, these apps have several limitations. First, most are limited to a single risk factor. For example, Text2Quit [15] is an interactive mHealth program that sends text messages to offer advice, support, and reminders about quitting smoking, and ASA24 [16] is an automated self-administered recall system for collecting information about food intake. Our app has the advantage of covering multiple risk factors (unhealthy eating habits, sedentary lifestyle, alcohol, and tobacco consumption). Second, most of these apps lack personalization, particularly for the recommendation of digital health interventions (SMS, email, use of connected weighing scales, etc). Personalization is important for ensuring the successful adoption of the recommendations delivered [40]. For example, it has been shown [42] that SMS-based interventions should not be recommended to individuals not familiar with the use of such messages. Costly digital health interventions should not be recommended to patients with financial difficulty, and mobile interactive voice responses should not be recommended if the subject is deaf or unable to respond to questions via a touch-tone phone [42]. Our app has the advantage of delivering free personalized digital health interventions adapted to patient profiles, as defined in the French national health recommendations. In the future, we plan to increase the degree of personalization by considering additional conditions with a potential impact on patient adherence to recommendations (eg, living conditions such as work or family constraints). The consideration of such conditions will require the development of new guidelines, with the help of a multidisciplinary group of experts.

### Areas for Improvement

In the future, we aim to incorporate new functionalities, suggested by the participants, into the app. First, some study participants suggested adding functions for tracing behavioral changes. These changes could be monitored with connected objects such as t-shirts, bracelet watches, and smart socks [43]. Connected objects are increasingly being used by individuals for the instantaneous tracking of physiological activities such as physical activity, sleeping, or healthy eating [44]. Such monitoring could be very useful for patient follow-up and for adapting recommendations over time. Furthermore, the tracking achieved with these connected objects can be very effective for encouraging people to change their behavior [45,46]. For these reasons, we plan to connect our app to such tracking devices. Second, some study participants suggested delivering daily messages to support behavioral changes, whereas others suggested adding the possibility to talk with an expert or coach. Alternatively, a conversational agent (or chatbot), a computer program designed to simulate human text or verbal conversations [47,48], could be added. Chatbots have already been used as an alternative to face-to-face counseling for assisting clinicians, supporting patients trying to change their behavior and for monitoring health conditions [49]. A recent literature review [50] showed that interventions including conversational agents for coaching people in a healthy lifestyle could be more engaging, although data concerning their efficacy remain inconclusive. We will incorporate a conversational agent into our app in the future to increase the chances of behavioral changes being successfully adopted over time.

### Conclusions

We developed Prevent Connect, an app based on national recommendations, to encourage behavioral changes for the prevention of CVDs. The app allows users to self-assess the 4 most important risk factors (unhealthy eating habits, sedentary lifestyle, alcohol, and tobacco consumption) and delivers personalized recommendations and digital health interventions to help individuals improve their behavior. The app was considered to be of good quality by a panel of potential future users, but further improvements are required. The impact of the app on behavioral changes remains to be demonstrated and will be assessed in future studies.

## Abbreviations

CVD: cardiovascular disease
mHealth: mobile health
uMARS: user version of the Mobile App Rating Scale
